# Age-dependent association of clonal hematopoiesis with COVID-19 mortality in patients over 60 years

**DOI:** 10.1007/s11357-022-00666-5

**Published:** 2022-10-03

**Authors:** Marta Del Pozo-Valero, Marta Corton, Rosario López-Rodríguez, Ignacio Mahillo-Fernández, Javier Ruiz-Hornillos, Pablo Minguez, Cristina Villaverde, María Elena Pérez-Tomás, María Barreda-Sánchez, Esther Mancebo, the STOP_Coronavirus Study Group, Lidia Fernández-Caballero, Ruth Fernández Sanchez, Inés García Vara, Laura Marzal Gordo, Andrea Martínez-Ramas, Lorena Ondo, Raquel Romero, Miguel Górgolas, Alfonso Cabello, Germán Peces Barba, Sara Heili, César Calvo, Arnoldo Santos, María Dolores Martín Ríos, Olga Sánchez-Pernaute, Lucía Llanos, Sandra Zazo, Federico Rojo, Felipe Villar, Raimundo de Andrés, Ignacio Jiménez Alfaro, Ignacio Gadea, Celia Perales, Antonio Herrero, Juan Carlos Taracido, Elisa García-Vázquez, Rubén Jara-Rubio, José A. Pons-Miñano, Juana María Marín-Martínez, María Teresa Herranz-Marín, Enrique Bernal-Morell, Josefina García-García, Juan de Dios González-Caballero, María Dolores Chirlaque-López, Alfredo Minguela-Puras, Manuel Muro-Amador, Antonio Moreno-Docón, Genoveva Yagüe-Guirao, José M. Abellán-Perpiñán, Jorge E. Martínez-Pérez, Fernando I. Sánchez-Martínez, Alberto Utrero-Rico, Mario Fernández-Ruiz, Octavio Carretero, José María Aguado, Rocío Laguna-Goya, Yolanda Cañadas Juárez, Ángel Jiménez, María Herrera Abián, Mercedes García Salmones, Lidia Gagliardi Alarcon, María Rubio Oliveira, Carlos Fabian Castaño Romero, Carlos Aranda Cosgaya, Virginia Víctor Palomares, Leticia García Rodríguez, María Sánchez Carpintero Abad, María Carmen García Torrejón, Estela Paz-Artal, Encarna Guillén-Navarro, Berta Almoguera, Carmen Ayuso

**Affiliations:** 1 Department of Genetics & Genomics, Instituto de Investigación Sanitaria-Hospital Universitario Fundación Jiménez Díaz, Universidad Autónoma de Madrid (IIS-FJD, UAM), Avenida Reyes Católicos 2, 28040 Madrid, Spain; 2 Centro de Investigación Biomédica en Red de Enfermedades Raras (CIBERER), Instituto de Salud Carlos III, Madrid, Spain; 3 Present Address: Department of Pharmaceutical and Health Sciences, School of Pharmacy, Universidad San Pablo-CEU, Madrid, Spain; 4 Department of Epidemiology, Instituto de Investigación Sanitaria-Hospital Universitario Fundación Jiménez Díaz, Universidad Autónoma de Madrid (IIS-FJD, UAM), Madrid, Spain; 5 Allergy Unit, Hospital Universitario Infanta Elena, Madrid, Spain; 6 School of Medicine, Universidad Francisco de Vitoria, Madrid, Spain; 7 Bioinformatics Unit, Instituto de Investigación Sanitaria-Hospital Universitario Fundación Jiménez Díaz, Universidad Autónoma de Madrid (IIS-FJD, UAM), Madrid, Spain; 8 Instituto Murciano de Investigación Biosanitaria Virgen de La Arrixaca (IMIB-Arrixaca), Murcia, Spain; 9 School of Health Sciences, Universidad Católica San Antonio de Murcia (UCAM), Murcia, Spain; 10 Department of Immunology, Hospital Universitario, 12 de Octubre, Madrid, Spain; 11 Instituto de Investigación Sanitaria Hospital, 12 de Octubre (imas12), Madrid, Spain; 12 Department of Immunology, Ophthalmology and ENT. School of Medicine, Universidad Complutense de Madrid, Madrid, Spain; 13 Centro de Investigación Biomédica en Red de Enfermedades Infecciosas (CIBERINFEC), Instituto de Salud Carlos III, Madrid, Spain; 14 Medical Genetics Section, Pediatric Department, Hospital Clínico Universitario Virgen de La Arrixaca, Murcia, Spain; 15 School of Medicine, Universidad de Murcia (UMU), Murcia, Spain

**Keywords:** COVID-19, Mortality risk, Clonal variants

## Abstract

Clonal hematopoiesis, especially that of indeterminate potential (CHIP), has been associated with age-related diseases, such as those contributing to a more severe COVID-19. Four studies have attempted to associate CHIP with COVID-19 severity without conclusive findings. In the present work, we explore the association between CHIP and COVID-19 mortality. Genomic DNA extracted from peripheral blood of COVID-19 patients (*n* = 241 deceased, *n* = 239 survivors) was sequenced with the Myeloid Solutions™ panel of SOPHiA Genetics. The association between clonality and age and clonality and mortality was studied using logistic regression models adjusted for sex, ethnicity, and comorbidities. The association with mortality was performed with patients stratified into four groups of age according to the quartiles of the distribution: 60–74 years, 75–84 years, 85–91 years, and 92–101 years. Clonality was found in 38% of the cohort. The presence of CHIP variants, but not the number, significantly increased with age in the entire cohort of COVID-19 patients, as well as in the group of survivors (*p* < 0.001). When patients were stratified by age and the analysis adjusted, CHIP classified as pathogenic/likely pathogenic was significantly more represented in deceased patients compared with survivors in the group of 75–84 years (34.6% vs 13.7%, *p* = 0.020). We confirmed the well-established linear relationship between age and clonality in the cohort of COVID-19 patients and found a significant association between pathogenic/likely pathogenic CHIP and mortality in patients from 75 to 84 years that needs to be further validated.

**Supplementary Information:**

The online version contains supplementary material available at 10.1007/s11357-022-00666-5.

## Introduction

Clonal hematopoiesis (CH) refers to the expansion of hematopoietic cells with the same acquired mutation [1], and is a common age-associated phenomenon in the general population [2–4]. Among the different types of CH, that of indeterminate potential (or CHIP) has received the most attention over the last decade [1–4]. CHIP denotes somatic mutations, both single nucleotide variants (SNVs) and indels, present with a variant allele fraction (VAF) of > 2% in genes involved in myeloid neoplasia in patients without overt hematological malignancy [2, 3].

Due to the significant increase of CHIP with age, its role in age-related diseases, such as cancers and cardiovascular diseases, and increased mortality has been the focus of active research [2–4]. The studies by Jaiswal and Genovese were the first to evidence an increased risk of mortality, and of hematological cancer, coronary heart disease, and ischemic stroke associated with the presence of somatic mutations [2–4]. The fact that such diseases are known risk factors for severe COVID-19, along with the pro-inflammatory effect that CHIP may have on myeloid cells [2, 3], has given rise to the hypothesis that CHIP-exacerbated inflammatory signaling may be associated with the severity of SARS-CoV-2 infection [5].

CHIP has been suggested to contribute to the cytokine storm syndrome associated with higher mortality from COVID-19 [6–9]. Since the beginning of the pandemic in March 2020, four studies have attempted to link the presence of CHIP to outcomes of COVID-19 disease [6–9]. The main outcome of such studies was severity, using different definitions such as hypoxia, intubation, intensive care unit (ICU) hospitalization, or death [6–9]. Only Bolton and colleagues were able to find a positive association between CHIP and disease severity [9]. Additionally, Shivarov & Ivanova suggested a linear age-related relationship between the presence of clonality with the risk for mortality in SARS-COV2-infected patients [10] using existing data on the age-related increase in clonality published by Jaiswal and Genovese [2, 3] and the age-related increase in worldwide crude mortality rate from COVID-19 per age group from different countries. Based on the linear correlation found plotting such data, the authors proposed that the presence of clonality might be associated with the risk of fatal outcomes in COVID-19–infected patients.

In this study, we aimed to determine if the presence of CHIP was associated with COVID-19 mortality, as the most severe outcome of the disease, in a cohort of 480 patients over 60 years infected with SARS-CoV-2.

## Patients and methods

### Selection of patients and case–control definition

Cases and controls were selected from the STOP-CORONAVIRUS cohort, a sample of 3500 COVID-19 positive patients recruited from March to November of 2020 from four hospitals in Spain: Hospital Universitario Fundación Jiménez Díaz (HU-FJD), Hospital Universitario Infanta Elena (HU-IE), and Hospital Universitario 12 de Octubre (HU-12O) in Madrid, and Hospital Universitario Virgen de la Arrixaca (HU-VA) in Murcia. Extensive clinical data were available from the SARS-CoV-2 infection, which was confirmed in all patients by PCR or serological tests, until February 2021. Clinical data were either manually collected or extracted from individual electronic medical records using big data/artificial intelligence and then reviewed and refined by four independent researchers. Clinical information included primary demographic data, comorbidities, COVID-19 status, and diagnostic methods; symptoms, laboratory findings, and information regarding treatments, hospitalization, ICU admissions, and outcomes; and related complications from COVID-19.

Cases were selected fulfilling the criteria: > 60 years of age and with an outcome of death because of the SARS-CoV-2 infection (deceased patients). Controls were patients who survived the infection (survivors) matched with cases by age and sex. Individuals with a history of hematologic cancer were excluded.

The study was approved by the research ethics committees (REC) of each center (CEIm HU-FJD, FJD-Biobank, ref. PIC087-20; CEIm HU-VA, IMIB-Arrixaca Biobank, ref. COVID-19 RMu; and CREC HU-12O). Because of the health emergency, an exception to the requirement for informed consent for this cohort was also approved by the REC of each center. Wherever possible, patients provided written or verbal informed consent to participate. All samples were pseudonymized and clinical data were managed according to the existing legislation and institutional requirements.

### Sequencing and variant analysis

Genomic DNA was extracted from peripheral blood using an automated DNA extractor (BioRobot EZ1, QIAGEN GmbH). Detection of CH variants was performed using the Myeloid Solutions™ Panel (MYS) (SOPHiA Genetics, Saint Sulpice, Switzerland) which has a sensitivity threshold of 2% for low-frequency variants.

The panel consists of 191 targeted regions with a total 48.7 Kb from 30 genes implicated in hematological malignancies, including the complete coding sequences of 10 of them (Table S1). Libraries of 96 multiplexed samples were prepared following the manufacturer’s instructions and further sequenced on an Illumina NextSeq® 500 using a Mid-Output v2.5 kit with 2 × 150 bp reads. Sequence alignment, base calling, and variant annotation for SNVs and CNVs were performed using a specific Blood Cancers pipeline in the SOPHiA DDM® platform (SOPHiA Genetics, Saint Sulpice, Switzerland), a commercial artificial intelligence (AI)-powered cloud-based software. The mean coverage was 4131X, with > 99% of the target regions being covered by a minimum depth of 1000X.

Variants were considered CHIP if they met the following criteria: (1) variant allele frequency (VAF) between 2% and 35% covered by at least 20 reads; (2) variant type: missense, frameshift, stop-gain, in-frame indel, and splice canonical sites; (3) minor allele frequency (MAF) < 1% in population databases (gnomAD, ExAC, 1000GP) and with a frequency < 2% in our cohort of patients (< 10 individuals) for non-recurrent pathogenic variants; and (4) variant not classified as benign or likely benign in ClinVar. Variants meeting the above criteria were classified into five categories: benign and likely benign variants; variants of unknown clinical significance (VUS); and likely pathogenic (LP); and pathogenic (P) variants. The variant classification was based on the Belgian next-generation sequencing guidelines for hematological and solid tumours [11] (https://www.compermed.be/cms/public/compermed/assets/49jde8lgaiasg840) and previous reports [12]. Variants were classified as LP or P if they were known pathogenic variants or had been described as pathogenic in ClinVar, COSMIC, the scientific literature, and/or the SOPHiA Genetics community at least by two users. In addition, we classified as LP novel variants located in known gene hotspots; in-frame indels in the bZIP domain of the *CEBPA* gene; and clear loss-of-function (LoF) variants in tumor suppressor genes (*TET2* and *ZRSR2*). Missense and in-frame variants and LoF variants in oncogenes (*DNMT3A*) not reported before were classified as VUS. Varsome [13] and Franklin (https://franklin.genoox.com) databases were used to aid in the variant classification. Only the last three categories were considered for the subsequent analysis.

### Statistical analysis

Categorical variables were expressed as absolute and relative frequencies and continuous variables as means and standard deviations (SD). The association of variants in specific genes in cases and controls was tested using the Chi-squared test or Fisher’s exact test.

The association between clonality and age was tested with age as a continuous variable in the entire sample and stratified by case/control status. The association between clonality and mortality was studied with clonality defined as the presence and number of CHIP, number of P/LP variants, number of VUS, and VAF. The associations between clonality and age and clonality and mortality were performed using logistic regression models adjusted for sex, ethnicity, obesity, cardiovascular disease, hypertension, and diabetes. Due to the presence of missing values in some variables, multiple imputations by the chained equation (MICE) method were applied. A total of 10 complete datasets were generated, and models were fitted on each one of them. Rubin’s Rules were applied to pool the regression coefficients and their standard errors, and to get confidence intervals and *p*-values [14].

Results were expressed as odds ratios (OR), 95% confidence intervals (95% CI) and *p*-values. The analyses were stratified by age groups defined by the quartiles of age distribution. For the association with VAF, the frequency was introduced as a continuous variable. All statistical analyses were performed using the R software v4.0.5.

## Results

### Description of the cohort of patients and clonal variants

The case–control cohort consisted of 241 cases over 60 years of age deceased because of COVID-19 (referred to as “deceased”) and 239 controls (referred to as “survivors”), matched by age and sex. Information on the demographic and clinical variables used for the cohort selection is summarized in Table 1. Mean ages and male representation were similar between both groups: 82.6 ± 10.7 and 81.6 ± 10.0 years and males represented 51.9% (125/241) and 56.5% (135/239) of cases and controls, respectively.

**Table1:** Demographics and comorbidities data in the cohort of 480 COVID-19 patients. For the comparison of age means, a Student *t-*test was used. Categorical variables were compared with a Chi^2^ test

Variables	All patients	Deceased	Survivors	*p*-value
Age (mean ± SD)	82.1 ± 10.4	82.6 ± 10.7	81.6 ± 10.0	0.306
Male	54.2% (260/480)	51.9% (125/241)	56.5% (135/239)	0.310
Europeans	92.9% (446/480)	94.2% (227/241)	91.6% (219/239)	0.274
Comorbidities				
Obesity	25.3% (81/320)	25.5% (38/149)	25.2% (43/171)	0.942
Cardiovascular disease	25.1% (110/438)	27.4% (61/223)	22.8% (49/215)	0.271
Hypertension	68.2% (317/465)	65.7% (151/230)	70.6% (166/235)	0.248
Diabetes	16.9% (75/445)	17.0% (38/223)	16.7% (37/222)	0.916

**Fig. 1:**
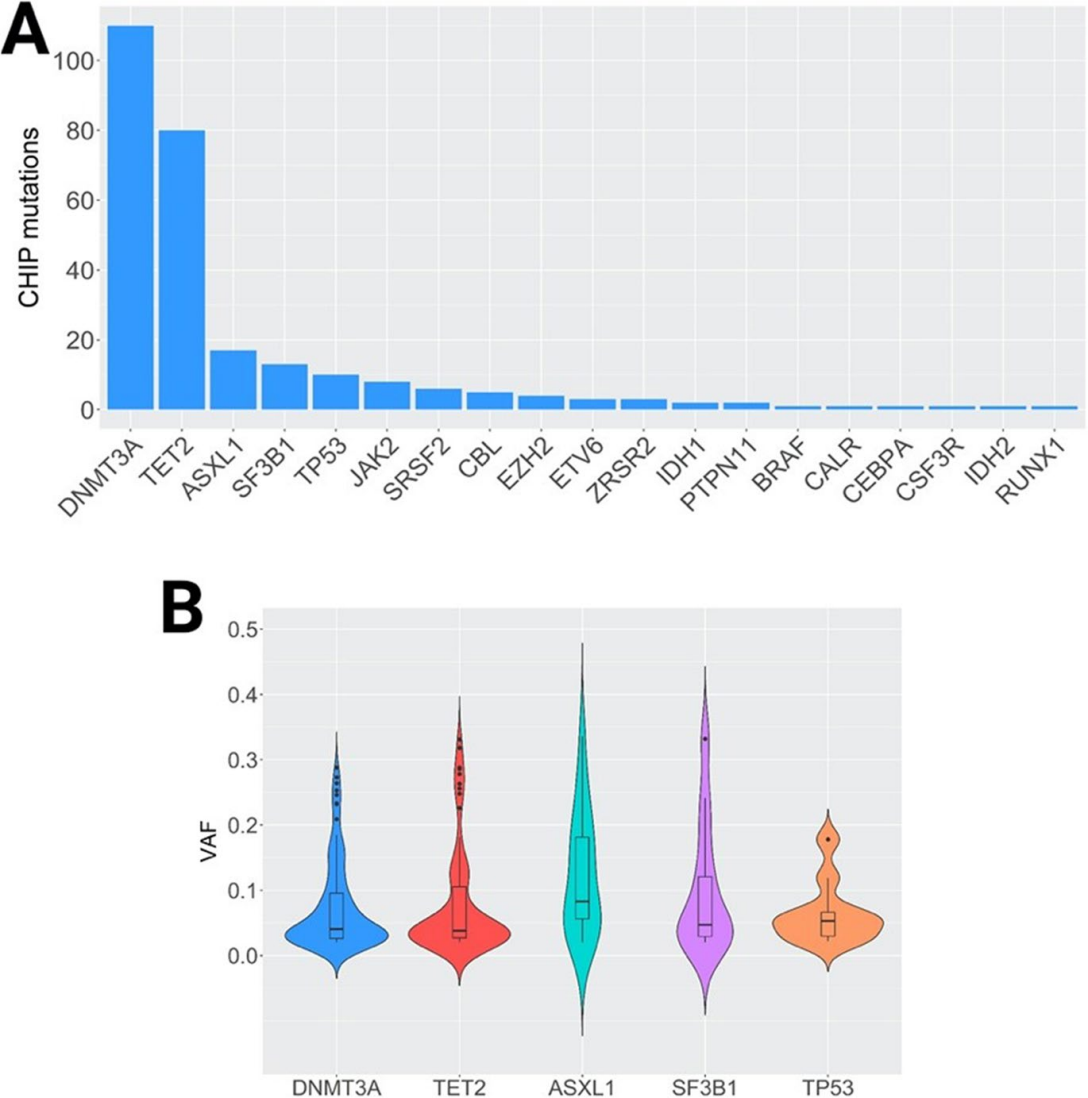
Mutational landscape of clonal variants in our cohort of COVID-19 patients. **A** Histogram representing the number of clonal variants per gene in the 19 genes where clonality was present. **B** Violin plots of VAF (y-axis) representing the distribution of variant allele frequencies (VAF) for clonal variants for the five most frequently mutated genes

Clonality, measured as the presence of at least one CHIP, was found in 182 patients (38% of the whole cohort), and the distribution was equal between cases and controls (91 deceased vs 91 survivors). These patients carried a total of 269 CHIP in 19 different genes (Fig. 1), with 14 being recurrent variants (Table S2). P/LP variants represented 61% of the total number of CHIP (*N* = 164; Table S2) and 39% were considered as VUS. Of the 182 patients with CHIP, 119 carried one (65%), 46 patients (25%) carried two; ten patients (5%) carried three, and seven patients (4%) carried four different variants.

The majority of the 269 variants identified (85.5%) were in five genes (Fig. 1). *DNMT3A* and *TET2* variants were the most frequent, with CHIP affecting 41% (*N* = 110) and 30% (*N* = 80) of our cohort of COVID-19 patients, respectively. Other recurrent genes affected by clonality were *ASXL1* (*N* = 17; 6.3%), *SF3B1* (*N* = 13; 4.8%), and *TP53* (*N* = 10; 3.7%).

The median VAF of CHIP was 4.4%, ranging from 2% to 34%. Of the 269 variants, 51.7% (139/269) had a VAF < 5%, 37.5% (101/269) a VAF between 5%, and 20% and 10.8% (29/269) a VAF ≥ 20%. The VAF distribution in the five most frequently mutated genes is shown in Fig. 1B. The frequency of CHIP (P/LP and VUS) in each gene was compared between deceased and survivors, but no significant association was found (data not shown).

### Significant increase of CHIP with age and of LP/P variants in deceased COVID-19 patients between 75 and 84 years

**Fig. 2:**
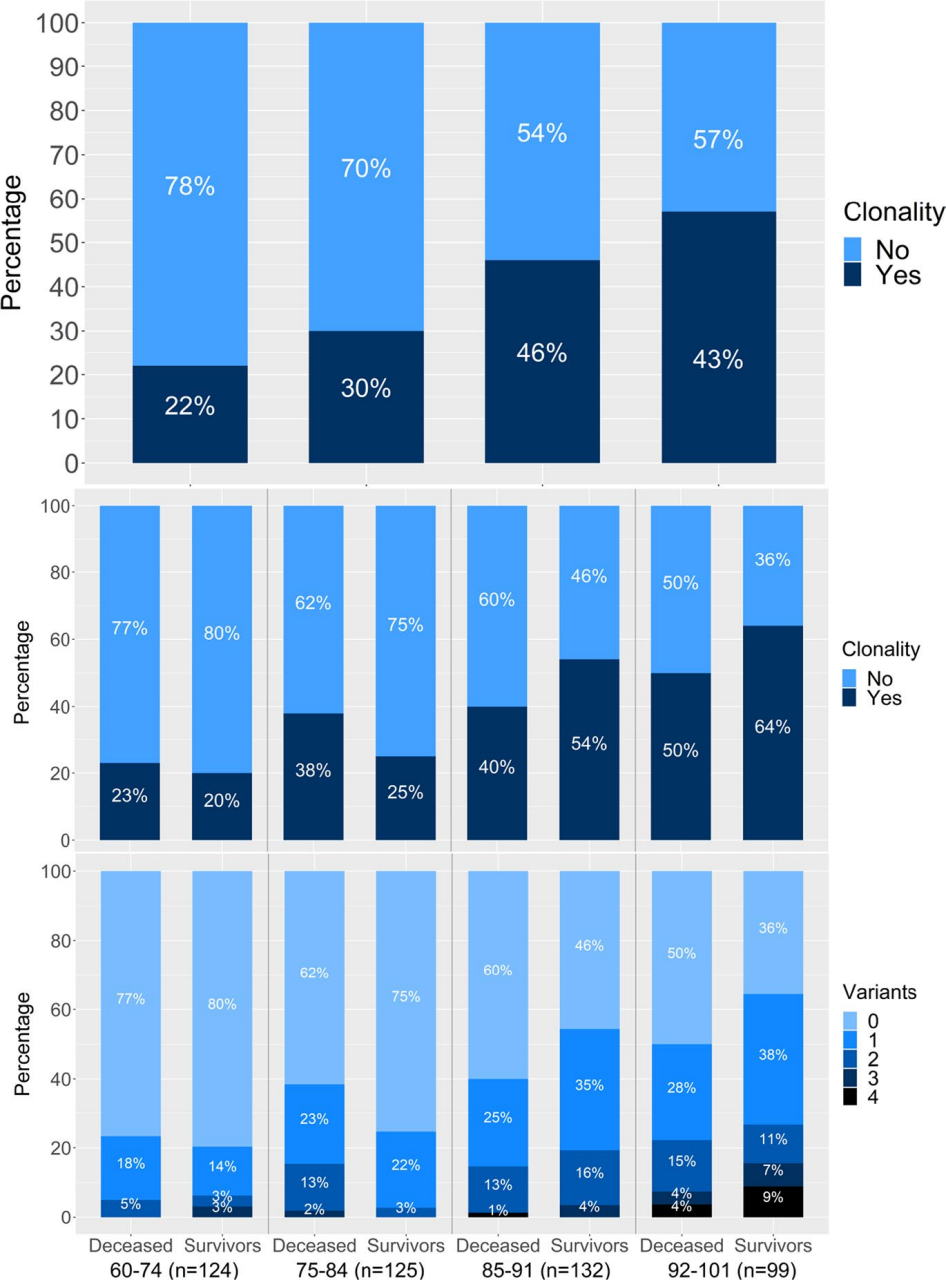
Clonality in COVID-19 patients. Frequency of CH in the entire cohort was stratified by age (**A**), presence (**B**) and the number of CHIP variants (**C**) in deceased patients and survivors in the same age groups

The association between clonality and age was tested using logistic regression models in the entire sample. For the association of clonality with mortality, patients were stratified by age into four groups based on the quartiles of the distribution of age: 60–74 years (*N* = 124; 60 deceased -48.4%-), 75–84 years (*N* = 125, 52 deceased -41.6%-), 85–91 years (*N* = 132, 75 deceased -56.8%-), and 92–101 years (*N* = 99, 54 deceased -54.5%-). The percentage of patients with CHIP per age group in the entire cohort, and the presence and number of CHIP in deceased vs survivors are illustrated in Fig. 2.

**Fig. 3:**
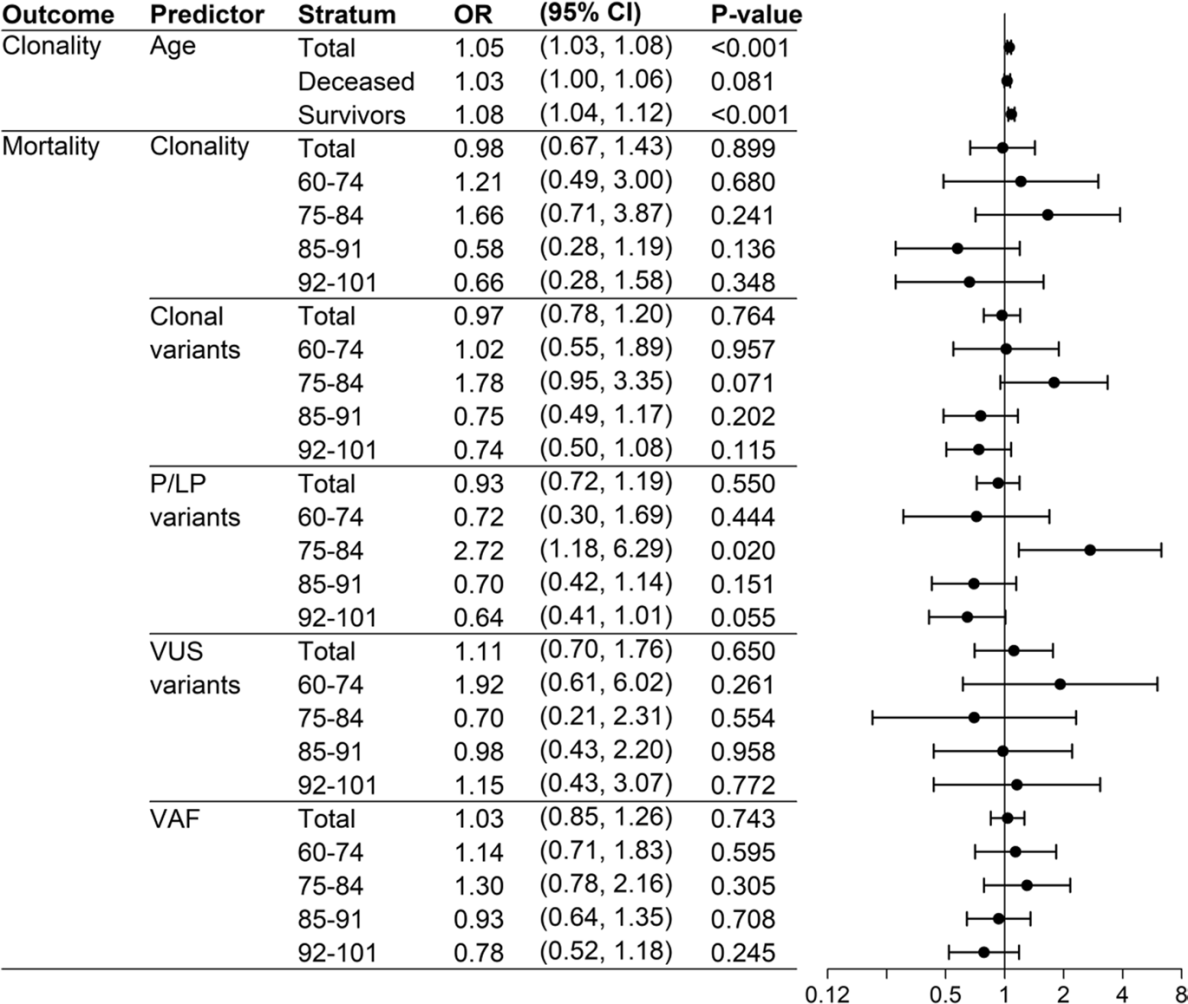
Odds ratios obtained by logistic regression for the association between CH and mortality of COVID-19 in our cohort, stratifying by age and adjusted by sex, ethnicity, obesity, cardiovascular disease, hypertension, and diabetes. Cardiovascular disease includes the following pre-existing conditions: ischemic heart disease, heart failure, cardiac arrhythmia and peripheral vascular disease. “Clonality” refers to the presence of clonal variants and “clonal variants” indicates the total number of clonal variants. In the association of clonality with age, age is introduced as a continuous predictor, and in the association between mortality and clonality, clonality is a dichotomous variable. Clonal variants, P/LP variants, VUS variants, and VAF are introduced as continuous predictors

The presence of CHIP, but not the number, increased with age (Fig. 2), and that association was statistically significant in the entire cohort of COVID-19 patients (OR = 1.05 (1.03–1.08); *p* < 0.001), as well as in the group of survivors (OR = 1.08 (1.04–1.12); *p* < 0.001). The association of clonality with mortality was assessed in COVID-19 patients stratified by age using a logistic regression adjusting for sex, ethnicity, obesity, cardiovascular disease, hypertension, and diabetes. The graphic representation of the adjusted logistic regression results is shown as a forest plot in Fig. 3. The distribution was not significantly different between deceased and survivors, except for the 75–84 age group (Fig. 3), where deceased patients had a significantly higher presence of P/LP variants compared to survivors (18 of 52 -34.6%- vs 10 of 73 -13.7%-, *p* = 0.020).

## Discussion

Based on the growing evidence of a higher incidence of age-related conditions and mortality with the presence of CHIP, and age being the strongest determinant of COVID-19 progression, there is currently an effort to link such genetic variation with worse outcomes of the disease [6–9]. In addition to aging, CHIP is suggested to sustain infectious-triggered inflammatory processes, both acute and chronic, such as those influencing outcomes of COVID-19 [2, 3]. CHIP has been associated with increased levels of C-reactive protein, chronic obstructive pulmonary disease [4, 15], and vasculitis [2, 16]. To date, four studies have explored the association of CHIP with COVID-19 severity defined by different outcomes, such as hospitalization, hypoxia, intubation and/or death; but only the work published by Bolton and colleagues in 2020 reported an increased risk of severe outcome [6–9].

In the present work, we confirmed the well-established linear relationship between age and clonality [2, 3], defined as the presence, but not the number, of CHIP in the entire cohort of COVID-19 patients, as well as in the group of survivors. CHIP was present in 38% of the 480 patients, and this frequency was similar in both deceased and survivors. Notably, the frequency of CHIP in our cohort is substantially higher than that reported in the studies led by Jaiswal and Genovese in the general population (10% in people older than 65 years and up to 18% in patients over 90 years, respectively [2, 3]) and also than frequencies reported in COVID-19 patients by Hameister and colleagues (25%) [8], Petzer and colleagues (19.4%) [7], and Bolton and colleagues (20% when the 89 genes overlapping the two cohorts studied were considered) [9]. Duployez et al. reported a frequency of 45%, which was higher than the frequency found in their control group, which consisted of a sample of patients with an unspecified hematological process for which a genetic study was conducted [6]. Age of participants, the definition of CHIP and the VAF threshold considered for clonal variants, the genes included in each study, and the coverage and analytical sensitivity of the technology used to detect clonal variants could account for the differences observed [2, 15].

An alternative explanation for the above differences is that the prevalence of CHIP could be higher in COVID-19 patients conferring a risk factor for the disease. We did not include a COVID-19 free control population and studies investigating the potential role of CHIP as a susceptibility factor for the infection have failed to find significant results [6, 8]. Nevertheless, these studies are either limited in their sample sizes [6, 8], or the controls have been recruited retrospectively, and the COVID-19 status is not stated or known [6]. Also, some of these controls are not healthy, selected based on existing sequencing data [6, 8, 9], and thus with the underlying disease phenotype potentially impacting the association. Therefore, further studies are needed to confirm or discard the role of CHIP in the susceptibility to COVID-19.

In line with previous reports [2, 3, 15, 17], the majority of CHIP identified were in five genes*,* with *DNMT3A* and *TET2* being the genes most frequently mutated (70%) followed by *ASXL1, SF3B1,* and *TP53.* However, no significant CHIP enrichment in a particular gene was found in deceased COVID-19 patients compared with survivors. To the best of our knowledge, a gene-specific association of CHIP in COVID-19 outcomes has neither been investigated [9] nor found significant [7, 8]. Only Duployez and coworkers found a significant association between *TET2* mutations and COVID-19 severity in males (OR = 3.940 (95% CI: 2.095–7.410, *p* < 0.001). However, the authors do not acknowledge this finding in their discussion and argue to have a limited sample size to draw a significant conclusion [6].

When patients were stratified by age and the analysis adjusted for sex, ethnicity, and comorbidities, we found an association between variants classified as P/LP, which were significantly more represented in the cohort of deceased patients from the group of 75–84 years compared with survivors in that age group (34.6% vs 13.7%, respectively). This result is opposed to that reported by Bolton et al., who found both non-putative cancer driver and silent variants significantly overrepresented in severe COVID-19 patients [9]. The authors described that such variants were found in non-recurrently mutated genes, the majority of which may not be considered CHIP genes, to the best of our knowledge. CHIP is defined as mutations that occur in a limited set of genes, which are well-known drivers of myeloid neoplasia [2, 3, 17]. Of the 37 genes reported by Bolton et al. to have non-PD variants, only *DNMT3A* was included in the myeloid gene panel tested in our cohort and we did not explore the effect of silent variation since the effect of such variants is difficult to ascertain.

It has to be noted that while this manuscript was under review, Zhou et al. reported no significant association between clonality and COVID-19 severity. The authors studied the effect of clonal variants in 56 genes implicated in CH in 568 patients aged 50–90 years, of whom 120 were mild or asymptomatic; 241 were hospitalized, and 207 were critically ill. The study found a frequency of CHIP ranging from 31% to 37% in COVID-19 patients, which is similar to what we report, and no significant differences between the three cohorts and the age groups [18].

A potential explanation for the significant association found in the 75–84 age group is that a gene-specific, age-dependent clonal expansion could influence worse outcomes. Buscarlet and coworkers in 2017 described an age effect for specific genes and found that *TET2* is the most prevalent mutated gene after 85 years of age. The authors hypothesized that *TET2* clones could be differentially favored with senescence [15]. A similar effect could be seen with other genes/age groups. It is also possible that along with genetic factors, other confounding variables could influence the differences observed. Patients were recruited during the first wave of the pandemic in 2020 when socio-sanitary factors also strongly influenced the disease outcome, such as limited access to hospitals, availability of respiratory aid devices, etc. Finally, a spurious association cannot be ruled out. Nonetheless, some limitations should be acknowledged. First, although the sample is larger than most studies performed to date in COVID-19, it may not be sufficient to identify significant associations. We cannot rule out that an undiagnosed or very early-stage malignant process may also be biasing this potential association, especially in those patients carrying more than one variant in CHIP genes. Our deep-sequencing panel, achieving high sensitivity, missed a few genes that were recurrent in other studies, but with very low prevalence.

The present study has several strengths that must be highlighted. It comprises a large, well-defined cohort of patients with COVID-19, with well-documented outcomes and extensive clinical data. Cases and controls have been selected based on the outcome of mortality, and controlled both by matching the overall cohort by sex and age and by adjusting the statistical analysis by known risk factors for COVID-19 severity such as sex and comorbidities like obesity, diabetes, hypertension, and cardiovascular disease. Our cohort is also one of the largest studied to date of patients deceased because of COVID-19 and enriched in patients older than 90 years old. Compared with other studies, cancer, or hematological malignancies at the time of recruitment were exclusion criteria.

In summary, we observed a high frequency of CHIP, mainly involving *TET2* and *DNMT3A* genes, in a cohort of COVID-19 patients. Although our findings cannot confirm a significant impact of CHIP on the fatal outcome of SARS-COV2 infection, a significant statistical association for increased odds was documented in patients from 75 to 84 years of age harboring clonal expansion in well-known pathogenic variants. This needs to be confirmed in an independent study.

## Supplementary Information

Below is the link to the electronic supplementary material.

Supplementary file1 (XLSX 39 KB)
